# Reconstruction of the Major Maternal and Paternal Lineages in the Feral New Zealand Kaimanawa Horses

**DOI:** 10.3390/ani12243508

**Published:** 2022-12-12

**Authors:** Muhammad Bilal Sharif, Robert Rodgers Fitak, Barbara Wallner, Pablo Orozco-terWengel, Simone Frewin, Michelle Fremaux, Elmira Mohandesan

**Affiliations:** 1 Department of Evolutionary Anthropology, University of Vienna, Djerassiplatz 1, A-1030 Vienna, Austria; 2 Human Evolution and Archaeological Sciences (HEAS), University of Vienna, Djerassiplatz 1, A-1030 Vienna, Austria; 3 Vienna Doctoral School of Ecology and Evolution (VDSEE), University of Vienna, Djerassiplatz 1, A-1030 Vienna, Austria; 4 Department of Biology, Genomics and Bioinformatics Cluster, University of Central Florida, 4110 Libra Dr, Orlando, FL 32816, USA; 5 Institute of Animal Breeding and Genetics, Veterinary University of Vienna, Veterinärplatz 1, A-1210 Vienna, Austria; 6 Cardiff School of Biosciences, Cardiff University, The Sir Martin Evans Building, Museum Avenue, Cardiff CF10 3AX, Wales, UK; 7 Feed2U Ltd., 19 Wairere Valley Road, Paparoa 0571, New Zealand; 8 InfogeneNZ (EPAGSC), School of Agriculture and Environment, Massey University, 1 Drysdale Drive, Palmerston North 4410, New Zealand

**Keywords:** feral horses, SNP genotyping, mitochondrial DNA, Y-chromosome, Kaimanawa, New Zealand, genomic resources, conservation value

## Abstract

**Simple Summary:**

New Zealand has the fourth largest feral horse population (Kaimanawa and Far North horses) in the world. The Kaimanawas (KHs) are feral horses descended from various domestic horse breeds released into the Kaimanawa ranges in the 19th and 20th centuries. Over time, the population size has fluctuated dramatically due to hunting, large-scale farming and forestry. Currently, the herd is managed by an annual round-up, limiting the number to 300 horses to protect the rare and unique native flora in this region. Here, we examined 96 KHs to investigate their genetic similarity with respect to other domestic horse breeds, using uniparental markers (mitochondrial DNA, Y-chromosome). Our results indicate that although six maternal and six paternal lineages contributed to the KH gene pool, the current population is dominated by few ancestral lineages, and possibly represents two KH sub-populations. We show that three horse breeds, namely Welsh ponies, Thoroughbred and Arabian horses had a major influence in the genetic-makeup of the extant KH population. Moreover, our results suggest that mitochondrial genetic diversity in KHs is closer to the Sable Island horses, and less than other feral horse populations around the world. Our current findings, combined with ongoing research will provide insight into the KH population-specific genetic variations and level of inbreeding. This will advance equine genomic research and improve the management strategies to conserve these treasured New Zealand horses.

**Abstract:**

New Zealand has the fourth largest feral horse population in the world. The Kaimanawas (KHs) are feral horses descended from various domestic horse breeds released into the Kaimanawa ranges in the 19th and 20th centuries. Over time, the population size has fluctuated dramatically due to hunting, large-scale farming and forestry. Currently, the herd is managed by an annual round-up, limiting the number to 300 individuals to protect the native ecosystem. Here, we genotyped 96 KHs for uniparental markers (mitochondrial DNA, Y-chromosome) and assessed their genetic similarity with respect to other domestic horses. We show that at least six maternal and six paternal lineages contributed unequally to the KH gene pool, and today’s KH population possibly represents two sub-populations. Our results indicate that three horse breeds, namely Welsh ponies, Thoroughbreds and Arabian horses had a major influence in the genetic-makeup of the extant KH population. We show that mitochondrial genetic diversity in KHs (π = 0.00687 ± 0.00355) is closer to that of the Sable Island horses (π = 0.0034 ± 0.00301), and less than other feral horse populations around the world. Our current findings, combined with ongoing genomic research, will provide insight into the population-specific genetic variation and inbreeding among KHs. This will largely advance equine research and improve the management of future breeding programs of these treasured New Zealand horse.

## 1. Introduction

Feral horse populations are found throughout the world [1]. They exist in a natural state and differ from their domesticated counterparts as they reproduce independently of human interventions. Feral horses represent valuable genomic resources that may be important for the future of horse breeding. Maintaining feral genetic diversity is especially important at this time when the genetic diversity of domestic horses (*Equus caballus*) is dramatically eroding and no truly wild horses exist in the world [2,3]. Although feral horse populations have been studied in their social organization, behavior and demography (reviewed in [4]), there is a lack of comprehensive and comparative genomic analysis on most feral horses. There are only three exceptional cases, in which comparative genome-wide SNP genotyping analyses were performed on feral Andean horses, American Mustangs [5], and the Canadian Sable Island and Alberta Foothills horses [6,7]. In these studies, the results were contrasted with various domestic breeds to understand genetic adaptation to high altitude [5], genetic diversity, phylogenetic relationships [6], and genomic consequences of inbreeding [7]. Except the studies mentioned above, most genetic studies on feral horses have been restricted to only a few populations from North America and often employ a limited number of specific genetic markers such as microsatellites and mitochondrial DNA (mtDNA) to investigate their genetic diversity and phylogenetic relationships to domestic horse breeds (e.g., [8,9,10,11,12]).

**Figure 1:**
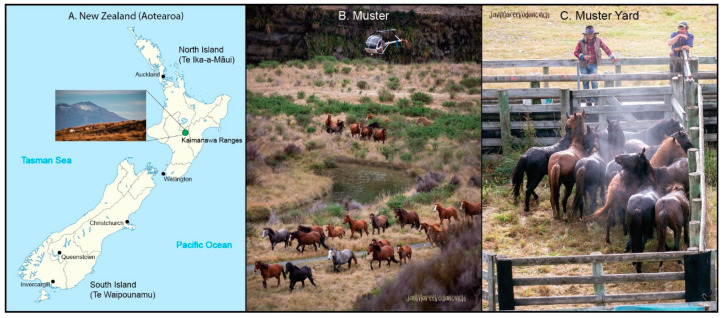
Geographical location and mustering process in Kaimanawa horses (KHs). (**A**) The location of the Kaimanawa mountain ranges, the habitat of feral KHs. (**B**) Annual mustering with helicopter, by which the horses are taken away from their natural habitat. (**C**) Mustering yard where animals are collected and examined by a veterinarian for their health and suitability for re-homing. *Image credit: Jan Maree Vodanovich*.

Currently, with an estimated number of 2,500 horses across the country, New Zealand’s feral horse population (Kaimanawa horses (KHs) and Far North horses) is the fourth largest in the world, after Australia (Brumbies; ~1 million), United States of America (Mustangs; ~120,000) and Canada (e.g., Sable Island and the Alberta Foothills; ~3500) [13]. Historically, domestic horses were first introduced to New Zealand in 1814 from New South Wales (AU) by Reverend Samuel Marsden [14]. Subsequently, more horses arrived with European travelers, settlers, explorers, and cavalry as well as those which were owned and traded by the Māori people (indigenous Polynesian people of mainland New Zealand) [14]. KHs were first seen and reported in the Kaimanawa mountain ranges located in central North Island of New Zealand in 1876 [14,15]. These feral horses descended from various domestic horse breeds released into this region in the 19th and 20th centuries (Figure 1A). Over the last 150 years, KHs have lived freely at altitudes up to 1,500 m above sea level in a unique ecological region devoid of predators and competitors. Over time, the population size fluctuated dramatically due to hunting, large-scale farming and forestry. By 1979, only 174 horses remained [16]. After this dramatic decline, the New Zealand Wildlife Act (1953) was amended to give KHs legal protection within a limited geographic region [14]. This protection resulted in a rapid population growth (10-fold in ~14 years) of horses in poor health conditions. The high stocking density consequently led to threatening the rare and unique native flora by trampling and overgrazing [17]. Since 1993, KH numbers have been controlled through systematic mustering (i.e., round-up) programs that keep the population size congruent with its botanical environment. The mustered horses were sent to slaughter or sold at auction. Today’s management strategies have been humanely improved and have been decided by the Kaimanawa Wild Horse Advisory Group. The herd continues to be managed by the New Zealand Department of Conservation, restricting the population census size to 300 individuals via annual helicopter mustering (Figure 1B,C).

The majority of mustered horses are offered to the public for rehoming, depending on their good health, suitability of the new home, and a taming plan having been put in place. To avoid further breeding in the domestic setting, and to facilitate the taming process, all young stallions and colts get castrated. Recently (May 2022), and after decades of research, immune-contraception was applied to 150 female mustered horses as an alternative and complementary option to rehoming for controlling the population growth [18]. This change has been marked as the start of a new era in the management of KHs, allowing the vaccinated mares, foals, and stallions to return to their home territories and re-form their family bands, rather than being broken apart and rehomed.

The KH’s uncertain origin beyond the historical reports raises questions regarding their phylogenetic relationship with other horse breeds, genetic diversity, long-term genetic health and viability. The first and only genetic study on KHs was conducted in the 1990s [19] and focused on comparing KH’s allele frequencies across 16 red blood cell and plasma protein markers with those in more populous equine breeds in New Zealand, i.e., pure-bred hot blood (Thoroughbreds, Arabians) and warm blood (Standardbreds, Station Hacks “work horses with no particular breed”), as well as a representative of a heavy cold blooded horse line (Shire Horse) as a more distant breed. The main conclusion was that KHs are not genetically homogenous. They more closely resembled to Thoroughbred and Station Hack horses that made up most of the military and farm horses of the early 20th century in New Zealand, than Shire Horses. Although this study was the first one to shed light on the genetic relationship of KHs to other domestic horse breeds, it lacks resolution in the phylogenetic tree due to the few numbers of markers. In addition, at the time of this study, KHs have been present in New Zealand for only ~10 generations, which is not an adequate amount of time for evolutionary forces to accumulate markedly different genetic variation between KHs and the breeds from which they developed.

Since the late 1990′s, no significant genetic studies have been conducted in KHs. Here, we infer for the first time the current maternal and paternal genetic diversity of KHs, by means of uniparental markers (mtDNA and Y-chromosome (Y-chr)). Previous studies on domestic horses have shown high levels of genetic diversity and lack of phylogeographic structure in mtDNA [3,20,21]. Although mtDNA is a powerful marker to study the contribution of various ancestral maternal lineages into a horse population, it is limited in terms of making a correspondence between mtDNA haplotypes (HTs), breeds and geography. In contrast, the paternally inherited Y-chr is an informative genetic marker for investigating the origin and influence of very recent paternal lineages. Here, for the first time, we use a combination of mtDNA and Y-chr markers to, (*i*) examine the number of mtDNA haplogroups/types (HGs/Ts) in KHs as an indirect indication of the population’s current genetic diversity, and (*ii*) determine Y-chr HG/T and compare them to the other domestic breeds to understand the origin and influence of different patrilines in the KH gene pool.

In addition, pictorial records obtained from the largest archival photos of the Kaimanawa horses, suggest the existence of two KH herds within the muster area [13]. The grey horses are exclusively documented in the southern zones, whereas liver chestnut horses are found more in northern zones [13]. Although, we have no geographic information from the mustered KHs (southern vs. northern zones), the coat color information was provided by KH owners. Here, we attempt to genetically investigate whether there is population structure within the documented area, using a combination of uniparental genetic markers and coat color information.

## 2. Materials and Methods

### 2.1. Sample Collection

We collected hair follicles from a total of 96 KHs (53 female, 43 males), mustered during the period of 1993–2019 from different zones in the Kaimanawa ranges and rehomed by private owners or sampled for research purposes (Table S1). Among which, there is one sample from a male animal mounted on display at the Auckland War Memorial Museum. To avoid any stress and discomfort to animals during sampling, the owners followed two main strategies: firstly, sampling was performed over the summer period when hair follicles are most relaxed, and secondly, only a few hairs were pulled out at any attempt, with several repeats to complete the sampling.

### 2.2. DNA Extraction and SNP Genotyping

Genomic DNA was extracted from hair follicles of 96 KHs, following a magnetic bead-based DNA extraction protocol by the technical personnel at Neogen. The individuals were genotyped with the Neogen GeneSeek Genomic Profiler (GGP) Equine v4. array (75K), following the manufacturer’s instruction. This array contains ~71K SNP markers evenly distributed throughout the equine genome (autosomal and X-chr: ~70,231, mtDNA: 1187 and Y-chr: 170). The raw data files from Illumina’s GenomeStudio Final Report were converted to plink input files (lgen, fam and map), using a custom python script. Following [22], genotypes with bad quality calls (GenCall score < 0.15) were marked as missing data.

### 2.3. Relatedness Calculation

Prior to relatedness estimation, the following SNPs and individuals were excluded using PLINK v1.90 [23]: (*i*) SNPs that were missing in >5% of individuals; (*ii*) SNPs with minor allele frequency < 5%; (*iii*) SNPs with Hardy–Weinberg equilibrium deviation (--hwe) with *p-value* < 1 × 10^−5^ (as recommend in [24,25]); (*iv*) SNPs that were in linkage disequilibrium (--indep-pairwise 50 5 0.5; randomly one SNP from each pair was removed); and (*v)* individuals with >5% missing data.

As pedigree information (first-degree relatives) was only available for a few samples, we performed the identity-by-descent (IBD) analysis to evaluate the relatedness of the individuals. We calculated the genome sharing value (pi-hat) based on autosomal SNPs, using PLINK v1.90 (--genome; [23]). The filtered dataset included 91 individuals and a total of 24,943 autosomal SNPs. Individuals with pi-hat ≥ 0.25 (second-degree relatives and closer) were further marked as related (Table S2).

### 2.4. Mitochondrial DNA Analysis

To evaluate mtDNA genetic diversity, we extracted 1187 mt-variants from the KH genotype data. The distribution of these SNP markers along the entire length of equine mtDNA is shown in Figure S1. To filter the mtDNA-variants dataset, we used PLINK v1.90 [23] to exclude: (*i*) individuals with missing data > 5%; and (*ii*) SNPs with missing data in more than one individual. We further excluded: (*iii)* maternally related individuals (pi-hat ≥ 0.25 & same mtDNA HT) (Table S2). Our final mtDNA dataset consisted of 1081 SNPs genotyped in 71 KHs (Table S3). Moreover, we constructed a comparative dataset from published complete mtDNA sequences (79 domestic and two Przewalski’s horses, NCBI accessions: JN398377-457) reported in [20] as well as from Donkey mtDNA (*Equus asinus*: NC_001788.1) (Table S3), by extracting the overlapping SNPs positions as explained in Supplementary Text.

The genetic diversity indices including the total number of haplotypes (h), total number of polymorphic (segregating) sites (S), average number of nucleotide differences (k), haplotype diversity (H_d_), nucleotide diversity (π) and theta estimator based on the segregating sites (θ_W_) were calculated by DnaSP v6.12.03 [26] (Table 1). In addition, to understand whether the nucleotide diversity estimation is biased in the presence of the missing data (e.g., SNP genotyping), we calculated this value based on the complete mtDNA sequences, as well as 1,081 extracted positions in the global modern horse dataset [20], and compared the values (Table 1, Figure S2).

**Figure 2:**
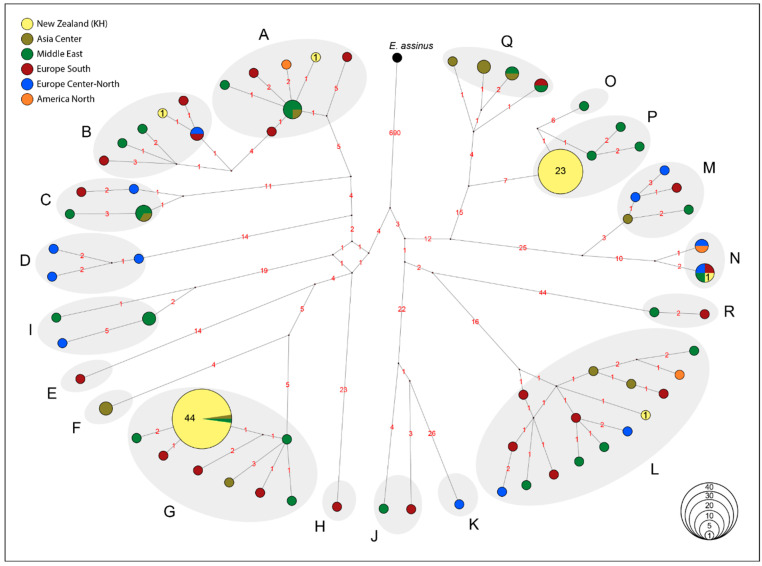
Median Joining network depicting mitochondrial DNA (mtDNA) haplogroup (HG) distribution (A–R) in KHs in relation to the global modern horses [20], based on 1081 SNPs. Haplotypes (HT) are indicated as circles, with size proportional to their frequency. The exact number of KHs are shown inside the circles. The colors indicate the geographical origin of the samples. The number of nucleotide differences between different haplotypes are shown on each branch. Donkey (*E. assinus*: NC_001788.1) mitogenome was used as an outgroup. The MJ network [28] was constructed using NETWORK v10.2.0.0 software (https://www.fluxus-engineering.com, accessed on 6 October 2022), by applying uniform substitution weights and without activating the external rooting option.

To determine the maternal phylogenetic relationship between KHs and other domestic horse breeds, we constructed a maximum likelihood (ML) tree using MEGA v.10.2.5 [27] with the TN93 (Tamura-Nei) as the best fit substitution model based on the Bayesian Information Criterion (BIC) (Figure S3). In addition, to investigate the genetic similarity between KHs and other domestic horses, a median-joining (MJ) network [28] was constructed, using NETWORK v10.2.0.0 software (https://www.fluxus-engineering.com, accessed on 6 October 2022), by applying uniform substitution weights and without activating the external rooting option (Figure 2). The use of MJ network in inferring evolutionary relationships has been criticized by [29], due to two major shortcomings. Firstly, it is a distance-based approach based on the overall similarity between two sequences without taking the evolutionary model of DNA sequences into consideration, and secondly, it lacks the evolutionary direction (no rooting). Therefore, in this study we only used this approach to show the uniparental haplotype distributional in KHs, when compared to already established phylogenetic tree in the global modern horse dataset. The mtDNA HG nomenclature was adopted from [20], and the frequencies of HTs were calculated by direct counting (Figure 2).

To compare the genetic diversity in KHs with the other feral horse populations, we calculated the diversity indices (Table S5) and constructed a MJ network (Figure S4), using available sequences from partial mtDNA D-loop (255-bp, containing 28 SNPs). The comparative dataset included seven feral horse populations namely Assateague (NCBI accessions: GU014400-02), Fluoride Cracker (NCBI accessions: AY997150-51, AY997192), Grand Turk (NCBI accessions: HQ593024-34), Mustang (NCBI accessions: AJ413746-822), Sable Island (NCBI accessions: HQ592901-13, HQ592951-56, HQ593035, HQ593063), Saint-Pierre et Miquleon (NCBI accessions: HQ592920-29, HQ593044) and Theodore National Park (NCBI accessions: MG761995-97) [10,12,30,31,32] (Figure S4).

### 2.5. Y-Chromosome Analysis

To investigate the paternal ancestry and evaluate the Y-chr HT diversity in the KH population, 170 genetic variants, which were previously described as “male-specific Y-chr (MSY) variants” by [33], were genotyped on the GGP Equine array, and analyzed in male KHs (*n* = 43). Out of 170 variants, 121 determine the HTs in the so-called “crown” group (detected in the majority of modern domestic breeds), while 49 define non-crown HTs (detected in Asian and some northern European breeds) [33,34]. Five additional variants (rAF, rDT, qCU, qCR and qW), which are not included in the current version (v4) of the Illumina GGP Equine array and provide resolution within the clade A of the crown group, were inferred by determining the allelic states with LGC KASP assays [35] (Tables S8 and S9), as described in [33]. The positions of all Y-chr variants in the GGP Equine array (v4) are according to the LipY764 (GCA_002166905.2) Y-chr assembly [33]. Information about MSY markers, variant type, indicative haplogroups, and ancestral and derived alleles is presented in Table S6.

To filter the KH GGP array Y-chr SNPs dataset, we used PLINK v1.90 [23] to exclude: (*i*) individuals with missing data > 10%; (*ii*) variants with heterozygous calls in males; and (*iii*) variants with missing data > 5%. The remaining missing calls were imputed by introducing the allelic states observed in the samples belonging to the same HG, following the method described in [36] (Table S6). We further excluded: (*iv)* paternally related individuals (pi-hat ≥ 0.25 & same Y-chr HT) (Table S2). Our final Y-chr dataset consists of 157 SNPs genotyped in 37 male KHs.

**Figure 3:**
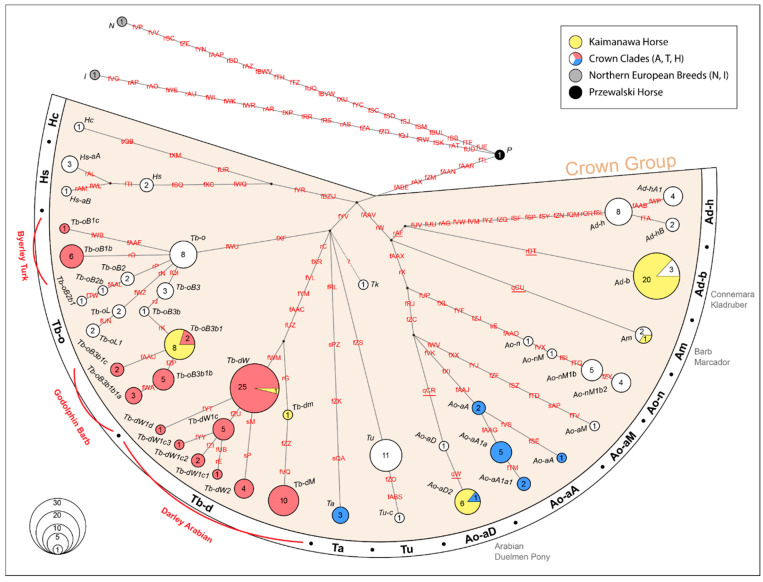
Median Joining network depicting Y-chromosome (Y-chr) haplogroup distribution in KHs in relation to the global modern horse breeds [34] based on 157 Y-chr SNPs. Haplotypes are indicated as circles and the numbers inside represent the number of individuals. The name of the variants observed in each haplotype are shown in red. The variants genotyped using KASP are distinguished by underline. The MJ network [28] was constructed using NETWORK v10.2.0.0 software (https://www.fluxus-engineering.com, accessed on 6 October 2022), by applying uniform substitution weights and without activating the external rooting option.

To investigate the phylogenetic relationship between KHs and other domestic horse breeds, a similar dataset of overlapping 157 SNPs was constructed from published Y-chr sequences (156 domestic, and one Przewalski’s horse, NCBI BioProjects accessions PRJNA430351 and PRJNA787432) reported in [34] (Table S6). A ML phylogenetic tree was generated using MEGA v.10.2.5 [27] with the K2 (Kimura-2) as the best fit substitution model based on the Bayesian Information Criterion (BIC) (Figure S5). Following the maximum parsimony trees constructed in [33,34], the topology and the number of horses exhibiting each Y-chr HT were visualized as a MJ network [28] generated with NETWORK v10.2.0.0 software (https://www.fluxus-engineering.com, accessed on 6 October 2022), using uniform substitution weights and without activating the external rooting option (Figure 3). As mentioned in Section 2.4, MJ network is only used to investigate the paternal haplotype distribution, and similarity between KHs and modern horse breeds without inferring phylogenetic relationships. The HG nomenclature was adopted from [34] and the frequencies of each HT were calculated by direct counting.

### 2.6. Population Structure and Genetic Distance within the KH Population

To evaluate the possibility of population structure within the sampled KHs, we performed nuclear (157 Y variants) F_ST_ (Fixation Index) [37] pairwise comparison, using ARLEQUIN v3.5.2.2 [38] with 1000 permutations. Since there is a lack of geographic information for the sampled KHs, we defined two sub-populations based on the observed dominant maternal HGs (G and P) (see results Section 3.2). In group-1, we included 27 male samples with maternal HG G, exhibiting paternal HGs Ad-b, Tb-oB3b1, Am, Ao-aD2 and Td-dm. In group-2, we included 13 male samples with maternal HG P, exhibiting paternal HGs Ao-aD2, Ad-b and Tb-oB3b1 (Table S10). We further cross checked our genetic data with phenotypic information (coat color) provided by KH owners (Table S10).

## 3. Results

### 3.1. Relatedness in KH population

Based on the IBD evaluation using autosomal SNPs, we show that ~53% of the individuals in the KH population have one or more second degree or closer relatives (pi-hat ≥ 0.25) in the sampled population. We excluded 22 maternally and 6 paternally related individuals from the mtDNA and Y-chr analysis, respectively (Table S2).

### 3.2. Mitochondrial DNA Genetic Diversity and Haplogroup Distribution

In this study, we successfully genotyped 1,081 mtDNA SNPs in 71 unrelated KHs (Table S3). We constructed a haplotype network to determine the genetic similarity between the KHs and the global mtDNA dataset representing 27 horse breeds across Asia, Europe, Middle East and the Americas [20]. KHs are clustered within six previously defined mtDNA HGs (A, B, G, L, N and P) (Figure 2, Table S4), with nucleotide diversity (π) = 0.025 ± 0.002, and haplotype diversity (H_d_) = 0.518 ± 0.043 (Table 1). The HGs G and P are the most frequent ones (G: 62%; P: 32%), while A, B, L and N were each represented by only single individuals (Figure 2, Table S4).

Furthermore, using the global modern horse dataset, our comparison between nucleotide diversity estimates using genotypic data (π = 0.042 ± 0.001) versus complete mtDNA sequences (π = 0.0049 ± 0.0002) indicate that in the face of missing data, the nucleotide diversity can be an overestimation of the true value as invariant sites are missing in SNPs genotype data and hence omitted from the calculations (Table 1, Figure S2).

In addition, we calculated the genetic diversity in KHs based on 28 SNPs retrieved from the partial mtDNA D-loop region (225-bp) and compared the value with the overlapping data from seven feral horse populations (Table S5). The mtDNA D-loop haplotype diversity in KHs (H_d_ = 0.11 ± 0.051) was comparable with the estimates obtained for the Sable Island (H_d_ = 0.095 ± 0.084) and Grand Turk (H_d_ = 0.182 ± 0.144) feral horse populations, while lower than other feral horse populations such as Assateague, Fluoride Cracker, Mustangs, Saint-Pierre et Miquleon and Theodore National Park. Moreover, our data show that even though the value of nucleotide diversity in KHs (π = 0.00687 ± 0.00355) is lower than any other feral horse populations, it is still closer to the Sable Island (π = 0.0034 ± 0.00301) population (Table S5). Among the feral horse populations, Saint-Pierre et Miquleon show the highest haplotype diversity (H_d_ = 0.782 ± 0.093) and nucleotide diversity (π = 0.12597 ± 0.01716) based on D-loop analysis. In addition, we observed slightly lower haplotype diversity and nucleotide diversity in a combined feral horse dataset (H_d_ = 0.714 ± 0.017; π = 0.0636 ± 0.002), compared to the domestic ones (H_d_ = 0.89 ± 0.02; π = 0.08166 ± 0.00586) (Table S5).

### 3.3. Y-Chromosome Genetic Diversity and Haplogroup Distribution

Our analysis on a combined dataset (157 SNPs) of male KHs (n = 37), and published Y-chr dataset (156 domestic horses and a single przewalski horse) retrieved from [34] (Table S6), indicate that KHs haplotypes are clustered within six previously defined MSY crown HTs (Ad-b, Am, Ao-aD2, Tb-dM, Tb-dW and Tb-oB3b1) (Figure 3, Table S7). All HTs detected in KHs have been previously reported in British ponies, Arabian and Thoroughbred horses.

The HT Ad-b is represented by more than half of our samples (Ad-b: 20/37), and it has been reported in breeds other than Arabians and Thoroughbreds, such as Connemara pony (Ireland) and Kladruber (Baroque type breed from Czech Republic). The HT Am is represented by only one KH sample (Am: 1/37), and it has been previously detected in Barb (North Africa) and Marchador (Brazil) horses. Among the three typical Arabian HTs (i.e., Ao-aA, Ta and Ao-aD2) commonly observed in occidental and local (Iran, Syria) Arabian lines, we only observed the Ao-aD2 in our KH samples (Ao-aD2: 6/37) (Figure 3).

Within the clade T, we have detected genetic signatures of two of the three influential Thoroughbred foundation sire lines in KHs, namely the Darley Arabian (HG Tb-d), and the Godolphin Barb (sub-HG Tb-oB3b1) [33]. Within the Darley Arabian line in KHs (2/37), the HT Tb-dW (Thoroughbreds) is represented by one KH, and another KH does not cluster with any HT defined by the samples reported in [34]. This sample contains the basal variant rG and have the reference allele for variants fZZ and fVQ (which are diagnostic to the HT Tb-dM), hence shown as HT *Tb-dm* here (Figure 3).

While the Darley Arabian line was represented by only two KHs, the Godolphin Barb line is more common (HT Tb-oB3b1: 8/37). We have not detected any signature of the sire line lineage related to Byerley Turk (sub-HG Tb-oB1), which has previously been reported in Thoroughbreds, Akhal Teke/Turkomans, and in a small set of Arabian horses with unknown ancestry [34].

### 3.4. Population Structure and Genetic Distance within the KH Population

We observed that among male horses with maternal HG G, the majority have British and Thoroughbred paternal ancestry (Ad-b: 18/27; Tb-oB3b1: 6/27), while the majority of KHs with maternal HG P have Arabian paternal ancestry (Ao-aD2: 7/13) (Table S10). F_ST_ pairwise genetic distance based on the Y-variants in KHs, indicate a significant (0.128, *p-value* < 0.05) and moderate structure within the male KH population harboring ancestral maternal G and P lineages. In addition, our results indicate that all grey KHs sampled in the Kaimanawa range (7/7) harbor maternal HG P, while chestnut horses (22/30) are predominantly found in horses with maternal HG G (Table S11).

## 4. Discussion

### 4.1. Genetic Diversity of Maternal Lineages in KHs

Although we observed the contribution of at least six ancestral maternal lineages in the KH gene pool, the majority of samples contributed to only two major HGs (G and P). These HGs are frequently observed in the Middle East and European horse breeds (Figure 2). The HG P has been previously found exclusively in samples from Middle East such as an unspecified Iranian breed, Caspian pony and Arabian horses, while HG G was found in central Asia (Akhal-Teke, Naqu), the Middle East (unspecified Iranian and Syrian horse, Arabian) and southern Europe (Giara horse, unspecific Italian breed) [20]. The unequal contribution of maternal lineages in today’s KHs can be explained by the expected effect of genetic drift in such a small, isolated population.

Mitochondrial haplotype and nucleotide diversity in KHs (H_d_ = 0.518 ± 0.043; π = 0.025 ± 0.002) is lower than the corresponding values obtained for the global modern horse dataset (H_d_ = 0.994 ± 0.003; π = 0.042 ± 0.001). This can, in principle, be explained by a smaller proportion of ancestral mitochondrial diversity in KH population, or by an effect of genetic drift in a small, isolated population. In addition, SNPs genotype data have ascertainment bias towards known variants in certain horse breeds. Therefore, any rare variants private to the KHs remain undetected. Although these unrepresented SNPs are more likely to be rare, they can be common in such a small, isolated population. In the absence of the complete mtDNA data, that bias leads to underestimating the diversity indices within the small, isolated KH population.

Furthermore, this genotyping bias limits the power of traditional statistical framework for neutrality test (e.g., Tajima’s *D* [39], and Fu and Li’s *F* [40]) to accurately evaluate the genomic signature of past evolutionary processes such as selection, demographic expansion or contraction. Therefore, we highlight the importance of performing whole genome sequencing in KHs and other feral horse populations to accurately infer the effect of past demographic events on the current genetic diversity of feral horses around the world, with greater application in management strategies.

Moreover, we attempted to compare the mitochondrial genetic diversity in KHs with other feral horses based on the overlapping partial D-loop region. However, it is worth noting that even though high levels of genetic diversity in horse mtDNA D-loop region (~350–650 bp) have been previously detected [21,32,41,42,43], recurrent mutations, tend to blur the structure of the phylogenetic tree as examined in [20] (Figure S4). As shown in [20] and confirmed by our mt-variants dataset, these few polymorphic positions (28 SNPs) retrieved from D-loop region are not informative enough to reconstruct the mtDNA haplotypes network (Figure S4), nevertheless they indicate the incorporation of a wide range of matrilineal lineages into the feral horses around the world.

### 4.2. Genetic Diversity of Paternal Lineages in KHs

We have observed an unequal contribution of at least six ancestral paternal lineages in the KH gene pool, dominated by three HTs (Ad-b, Ao-aD and Tb-oB3b1) (Figure 3). Regardless of this unequal paternal ancestry, all detected HTs belong to the crown group, which is a recently expanded horse MSY HG, predominant in modern domestic breeds. This confirms the historical reports indicating that the KH population was recently established from various modern domestic breeds imported to New Zealand, such as British ponies, Arabian horses and Thoroughbreds. Thus, beside the Thoroughbred ancestry shown in [19], our data add and confirm the historical contribution of imported horses with British and Arabian genetic influences in today’s extant KHs.

Based on written reports by James Boyd in the mid 1990’s [15], one of the main founders of the KH population were the Exmoor ponies which were imported to the Hawkes Bay area in New Zealand by Major George Gwavas Carlyon in the middle 19th century. These ponies were crossed with local horses resulting in the Carlyon Pony breed. The Carlyon ponies were later crossed with two Welsh pony stallions, which were imported by Sir Donald McLean, and a breed known as the Comet was produced. Apparently in the 1870s, McLean released a Comet stallion and several mares into the Kaingaroa Plains where KHs were roaming [15,19]. As Ad-b has been reported in Welsh ponies [44], our data support the major historical influence of these two Welsh ponies into KH male gene pool.

Considering all British and Irish pony breeds share a very similar breeding and lineage history—originated from Celtic ponies and were refined with oriental, Iberian and English Thoroughbred stallions [45]—we suggest that Exmoor ponies might exhibit the Ad-b, alike other British pony breeds. However, with no available information on the MSY variations in Exmoor ponies, their influence in KHs remains unresolved.

Among the three typical Arabians HGs/Ts (Ao-aA, Ta and Ao-aD2) reported in [34], we observed the HT Ao-aD2 in our KHs (Figure 3). When it comes to the origin of the Arabian line in KHs, it is documented that it was not until the l920’s that Arabian horses were first introduced into New Zealand, from India [46]. In addition, it has also been reported [15] that Nicholas Koreneff released an Arabian stallion into the Argo Valley region during the 1960’s, when he was forced to sell his farm. Since there is no information on whether this stallion was the only Arabian horse released in the Argo area, we cannot conclude that Ao-aD2 comes from this stallion. Nevertheless, our data for the first time demonstrate the influence of Arabian horses into the KH gene pool.

In addition to the Arabian and British pony breeds stated above, KHs show footprints of two of the three influential Thoroughbred foundation sires, namely the Darley Arabian (HG Tb-d) and the Godolphin Barb (sub-HG Tb-oB3b1) [33]. This major influx of Thoroughbreds or Thoroughbred descent horses into the KH population could be explained by a historical strangles epidemic which threatened the horses at the Waiouru Mounted Cavalry Stables in 1941 [14]. Some of these military horses died of the infection, whilst others survived and joined the feral KH herd.

These diverse and unequal contributions of different paternal lines in KHs can be the result of founder events, selection and genetic drift in a small, isolated population. Moreover, it can be explained by the natural social structure in horses, which is remarkably uniform in feral horses around the world [4,47,48]. There are stable breeding groups called “bands”, each containing a single stallion with one or more mares and their offspring. Mares and stallions within bands are unlikely to be closely related as both male and female offspring disperse from their natal band [4,47,48]. Other stallions live as bachelors alone or in groups with no breeding mares or juvenile females in mixed sex peer groups [4,47,48]. Therefore, the mating success of the ancestral foundation stallions would determine the proportion of the HGs observed in today’s KH population. Lastly, a consequence of this behavioral limitation is the further reduction of genetic variation that exacerbates the small effective population size that uniparental markers have. Consequently, if the effective population size is strongly reduced there is also a limit to the time in the past about which these markers have phylogeographic information (e.g., [49,50]). However, while these uniparental markers are relevant to establish sex biased evolutionary processes, exploiting the wealth of genealogical history in the nuclear DNA (e.g., via SNP arrays, ddRAD or whole genome sequencing) should be the next step to clearly understand the evolutionary origin of the KH.

### 4.3. Loss of Maternal and Paternal Lineages in the KH Population

We predict that the rare mtDNA HGs in KHs, such as A and B, will be lost in a few generations as they are observed only in one 14-year- and 20-year-old female, respectively (Table S10). In addition, we have only detected HGs L and N in two advanced age male individuals (18- and 16-year-old) (Table S10). Therefore, we conclude that either the maternal HG L and N has been already lost in the population, or they are in a very low frequency, and hence more susceptible to being lost in future as a result of genetic drift.

It is worth noting that maternal HG L, and paternal Tb-dw—which are common worldwide—were both observed only once and as a combination in one 18-year-old male individual with tan coat color. Tan coat color is very rare in the KH population and has been observed only in this sample in our collection. To reduce the sampling bias, we have almost sampled 32% of the KH population mustered during the period of 1993–2019 from different zones in the Kaimanawa ranges. Considering the small census size (~300 individuals) of the population and high level of relatedness (~50%), we can assume that our data exhibit a realistic picture of the maternal and paternal haplotype contributions in the KH population. Nevertheless, we cannot fully rule out the possibility of existing very rare and hence undetected haplotypes in the KH population.

### 4.4. Population Structure within KH Population: Parallel Contribution of European and Middle Eastern Parental Ancestry

We observed that among male horses with maternal HG G, the majority have British and Thoroughbred paternal ancestry, while the majority of KHs with maternal HG P have Arabian paternal ancestry (Table S10). This co-occurrence suggests a parallel contribution of these combinations of parental lineages, which can be explained by the effect of sampling from particular regions in the Kaimanawa ranges where initially different escaped and/or imported horses with European (G; Ad-b and Tb-oB3b1) and Middle Eastern (P; Ao-aD2) origins were settled. Although we have no geographical information from the sampled KHs (northern vs. southern), we further evaluated these results by adding phenotypic information (coat color) provided by KH owners. In agreement with pictorial evidence, our combined genetic and phenotypic data suggest the existence of two genetically isolated sub-populations in the Kaimanawa mountain ranges. There is one in the southern zones, where grey horses are exclusively found, and one in the northern area, where the population is dominated by bay and chestnut horses.

Whether this ancestral parental combination is only a reflection of the past founder groups, or today’s KH population splits into two genetically isolated sub-populations, would have a major impact on effective population size and genetic viability of the population. In the case of the later, it would not be a one herd of 300 horses, but rather an equal split of each approximately 150, which has to be carefully considered in management strategies to control the population size. Obtaining whole genomic data, and geographic information of sampled KHs would be the crucial future step to accurately evaluate the population structure of today’s KHs and to conduct informed management decisions.

## 5. Conclusions

In summary, our data indicate that although a diverse genetic ancestry contributed to the KH gene pool, today’s population is dominated by few maternal and paternal lineages. Therefore, to ensure the long-term viability of a genetically healthy population, minimizing the loss of the current genetic diversity in KHs must be a priority. This has already been initiated by returning the KHs with more rare and interesting colors (e.g., palomino, grey, tan) into the range, and excluding such mares from the immune-contraception intervention [9]. In addition, our genetic data in combination with phenotypic information provide support for the possibility of the existence of two genetically isolated sub-populations (northern vs. southern) of feral horses in the Kaimanawa mountain range.

Whether KHs harbor rare and population-specific (private) functional variations as a result of adaptation to their natural environment, can only be confirmed or rejected with more comprehensive genome-wide data. As previously shown [51], whole genome sequencing holds great potential to detect a large number of novel variants, many of which might be specific to the population under study. Ongoing genomic research on KHs would shed light on their genomic variation enabling them to adapt to their unique and harsh environment, and investigate the genomic footprints of past demographic events and future conservation strategies in this population.

## Figures and Tables

**Table 1:** Mitochondrial DNA (mtDNA) genetic diversity indices calculated for the KHs and the global modern horse dataset [20] based on the 1081 SNPs and complete mtDNA sequences (16,600 bp). The standard deviation values are shown in italics.

Genetic Diversity Indices	Kaimanawa Horse	Global Modern Horse [20]	
	1081 SNPs	1081 SNPs	Complete mtDNA
Number of samples (N)	71	81	81
Number of polymorphic (segregating) sites (S)	126	334	659
Number of haplotypes (h)	6	66	79
Average number of nucleotide differences (k)	27.3	45	83
Haplotype diversity (H_d_)	0.518 *(0.043)*	0.994 *(0.003)*	0.999 *(0.002)*
Nucleotide diversity (π)	0.025 *(0.002)*	0.042 *(0.001)*	0.0049 *(0.0002)*
Theta estimator based on the segregating sites (θ_W_)	0.024 *(0.006)*	0.062 *(0.016)*	0.008 *(0.002)*

## Data Availability

mtDNA and MSY SNPs for KHs, and comparative datasets are provided in Supplementary Tables S3 and S6, respectively.

## Supplementary Materials

The following supporting information can be downloaded at: https://www.mdpi.com/article/10.3390/ani12243508/s1, Figure S1: The coverage of mitochondrial SNP markers available on the GGP Equine v4. array (75K); Figure S2: Nucleotide diversity variation along the length of horse mtDNA shown for global modern horses and KHs; Figure S3: mtDNA ML phylogenetic tree constructed for KHs and global modern horses; Figure S4: Partial mtDNA D-loop (225 bp) HG network in KHs in relation to modern domestic and feral horses; Figure S5: Y-chr ML phylogenetic tree constructed for KHs and global modern horses; Table S1: Sample information of New Zealand feral KHs collected for this study; Table S2: Information on the relatedness among KHs; Table S3: Information on 1081 mtDNA SNPs genotyped in KHs and retrieved from global modern horses; Table S4: mtDNA HG affiliation in KHs and global modern horses; Table S5: Partial mtDNA D-loop (255-bp) genetic diversity in KHs compared with other feral horse populations; Table S6: Information on 157 MSY variants genotyped in male KHs using Equine GGP array and KASP genotyping techniques, and retrieved from global modern horses; Table S7: MSY HG affiliation in male KHs and global modern horses; Table S8: KASP Experimental setup used for genotyping five MSY variants in KHs; Table S9: Cluster plots for the five MSY variants genotyped using Bio-Rad CFX Manager 3.1; Table S10: Summary of mtDNA and Y-chr HG affiliation in male KHs, including their age at the time of sampling (~2020); Table S11: The number of KHs with different coat colors in relation to mtDNA and Y-chr HGs. References: [52,53,54].
