# Supplementary material for: Reconstruction of the Major Maternal and Paternal Lineages in the Feral New Zealand Kaimanawa Horses
Source: Animals (Basel). 2022 Dec 12;12(24):3508. doi: 10.3390/ani12243508 (PMC9774138; doi:10.3390/ani12243508)
Supplement: Supplementary file 1 [file animals-12-03508-s001.zip › animals-1984019-Supplementary.pdf]

Supplementary Materials

For

Reconstruction of the Major Maternal and Paternal Lineages in the Feral New Zealand Kaimanawa Horses

1. Mitochondrial DNA Analysis

1.1. Preparation of the comparative mitochondrial dataset

In order to extract 1,081 overlapping SNPs between KH and the global modern horse dataset [20] as well as donkey reference genome (NC_001788.1), we applied the following steps. The selected 1,081 nucleotide positions in the horse mtDNA reference genome (NC_001640.1) were converted to uppercase letters, using an in-house python script. The modified reference mitogenome was then aligned against 81 complete horse mtDNA sequences and donkey reference genome using MAFFT v7.427 [52] and the uppercase status of the SNPs was preserved (--preserve). The sequence alignments were visually inspected, using BioEdit v7.2.5 [53]. The new positions of the uppercase bases (due to insertions and deletions) in the reference mitogenome were marked and the corresponding bases were extracted for every sample (Table S3). Firstly, we tested and confirmed that these 1,081 overlapping mt-variants were sufficiently diagnostic to recapitulate the 18 major mtDNA HGs (A-R) reported in [20]. Secondly, for each sample we generated concatenated fragments from these overlapping SNPs, and inferred the phylogenetic relationship between KHs and the global modern horses (Figure 2,S3).

2. Y-chromosome Analysis

2.1. KASP Genotyping

KASP genotyping was performed on a CFX96 Touch™ Real-Time PCR Detection System, following the manufacturer's instruction. As positive control, male samples with known allelic states were included in each assay (Table S8). As negative control, genomic DNA from a female horse and water was included in each experimental set up following [34]. The cluster plots were analyzed by Bio-Rad CFX Manager 3.1 (Biorad, Vienna, Austria) (Table S9). Positive and negative samples for KASP genotyping assay were provided by Barbara Wallners' laboratory.

2.2. Excluding the fTE variant

We excluded the Y-chr variant (fTE: LipY764_contig76:14966) from our dataset, due to sequence discrepancies between the GGP array probes and the ones reported in [34]. We confirm that excluding this variant did not affect the HG affiliation in KHs, since it has only been detected in derived form in Icelandic horses [34] and thus is not required to explain the diversity observed within the crown group where all of our samples are placed.

2.3. MSY variants called in female individuals

Among 157 MSY variants previously reported in [34], we called 28 of them in different number of female individuals (between 3-51) (Table S6). This can be explained either by genotyping error in these female individuals, or "non-MSY" nature of these variants due to the complexity of Y-chr assembly. Nevertheless, these variants passing our filtering quality criteria (GenCall score > 0.15) were retained in male individuals and further used for phylogenetic analysis following [34].

3. Supplementary Tables

Table S1: Sample information of New Zealand feral KHs (n=96) collected for this study.

Table S2: Information on the relatedness among KHs. In case the related individuals harbor the same haplogroup (HG), one sample in each pair was retained (green color), and one (orange color) was removed from data analysis.

Table S3: Information on 1,081 mtDNA SNPs genotyped in KHs (n=93) and retrieved from the global modern horse dataset (n=81) [20].

Table S4: mtDNA HG affiliation in KHs (n=93) and the global modern horse dataset (n=81) [20].

Table S5: Partial mtDNA D-loop (255-bp) genetic diversity in KHs compared with other feral horse populations.

Table S6: Information on 157 MSY variants genotyped in male KHs (n=43), using Equine GGP array and KASP genotyping techniques, and retrieved from the global modern horse dataset (n=157) [34].

Table S7: MSY HG affiliation in male KHs (n=43) and global modern horse dataset (n=157) [34].

Table S8: KASP Experimental setup used for genotyping five MSY variants in KHs.

Table S9: Cluster plots for five MSY variants genotyped in KHs using Bio-Rad CFX Manager 3.1.

Table S10: Summary of mtDNA and Y-chr HG affiliation in KHs (n=93), including their age at the time of sampling (~2020).

Table S11: The number of KHs with different coat colors in relation to mtDNA and Y-chr HGs.

4. Supplementary Figures

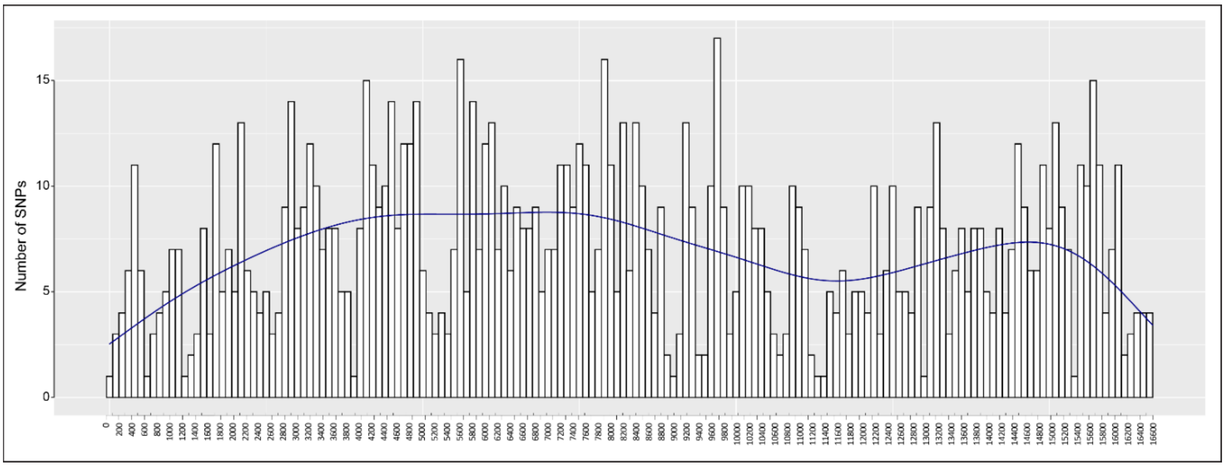

Figure S1: The coverage of the mitochondrial SNP markers available on the GGP Equine v4. array (75K) shown as the number and density of SNPs in windows of 200bp along the entire length of equine mtDNA (NC_001640.1)

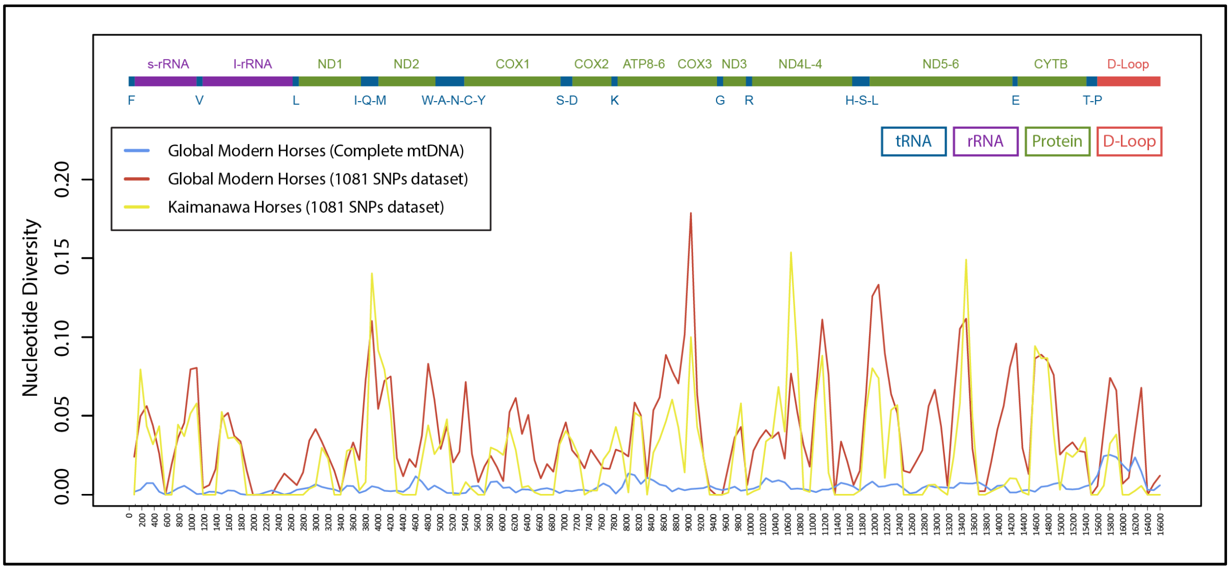

Figure S2: Nucleotide diversity variation along the length of mtDNA calculated in three different datasets i.e. Global Modern Horse [20] (complete mtDNA and 1,081 variants selected in this study) and KHs (1,081 variants), by considering windows of 200 bp (step size = 100), using DnaSP v6.12.03 [26]. The genetic map of the mitochondrial genome is presented on the top.

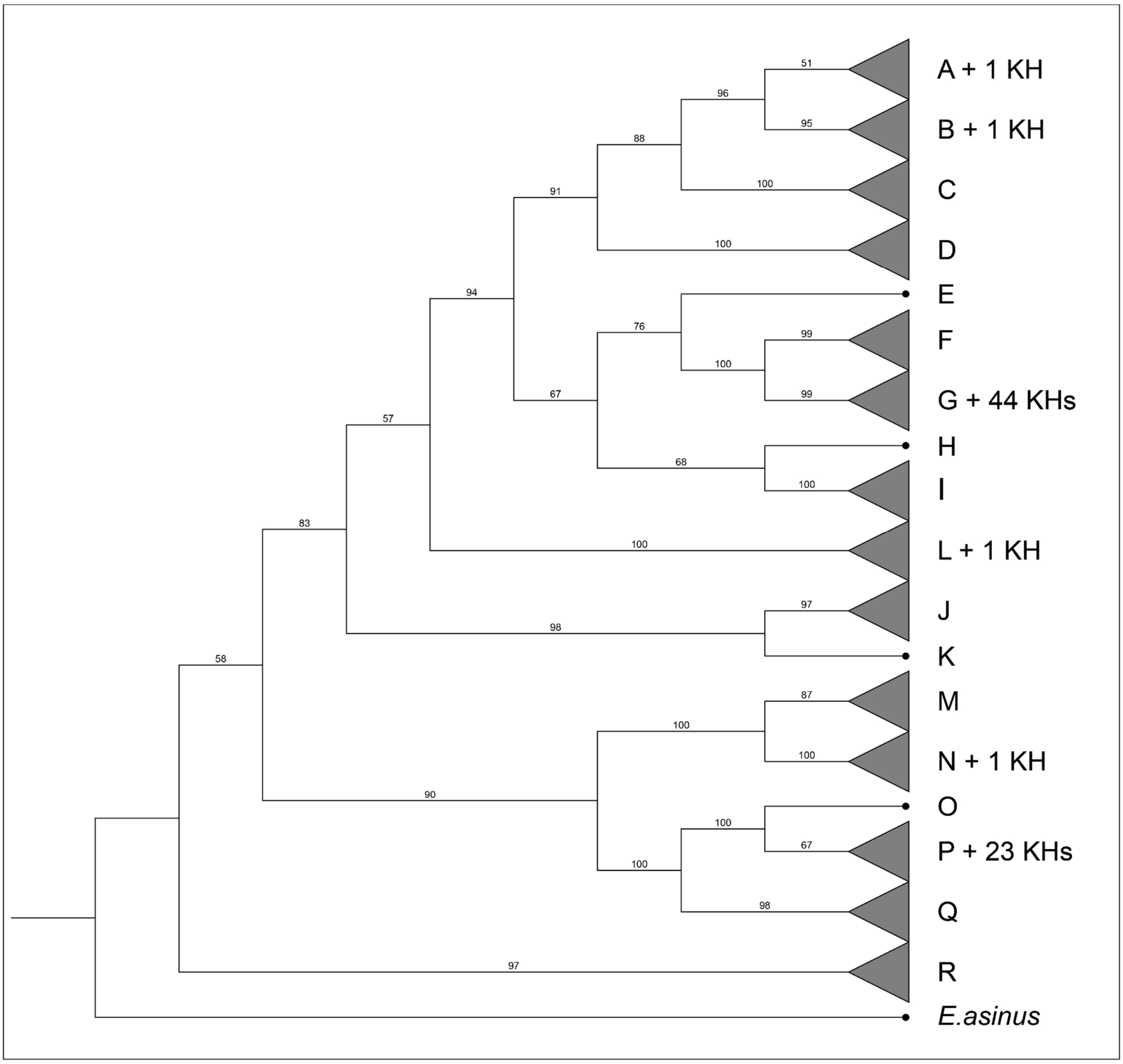

Figure S3: ML phylogenetic tree based on 1,081 mtDNA SNPs genotyped in KHs (n=71), and retrieved from the global modern horse dataset (n=81) [20]. The numbers on the branches indicate the bootstrap support, calculated using 1,000 replicates. The nomenclature of 18 major mitochondrial HGs (A-R, adopted from [20] and the number of KH individuals represented by each HG are shown to the right. Donkey's mitogenome (*E. assinus*: NC_001788.1) was used as an outgroup, and the tree was visualized using iTOL v6.4.2. [54]. ML phylogenetic tree was constructed using MEGA v.10.2.5 [27] with the TN93 (Tamura-Nei) as the best fit substitution model based on the Bayesian Information Criterion (BIC).

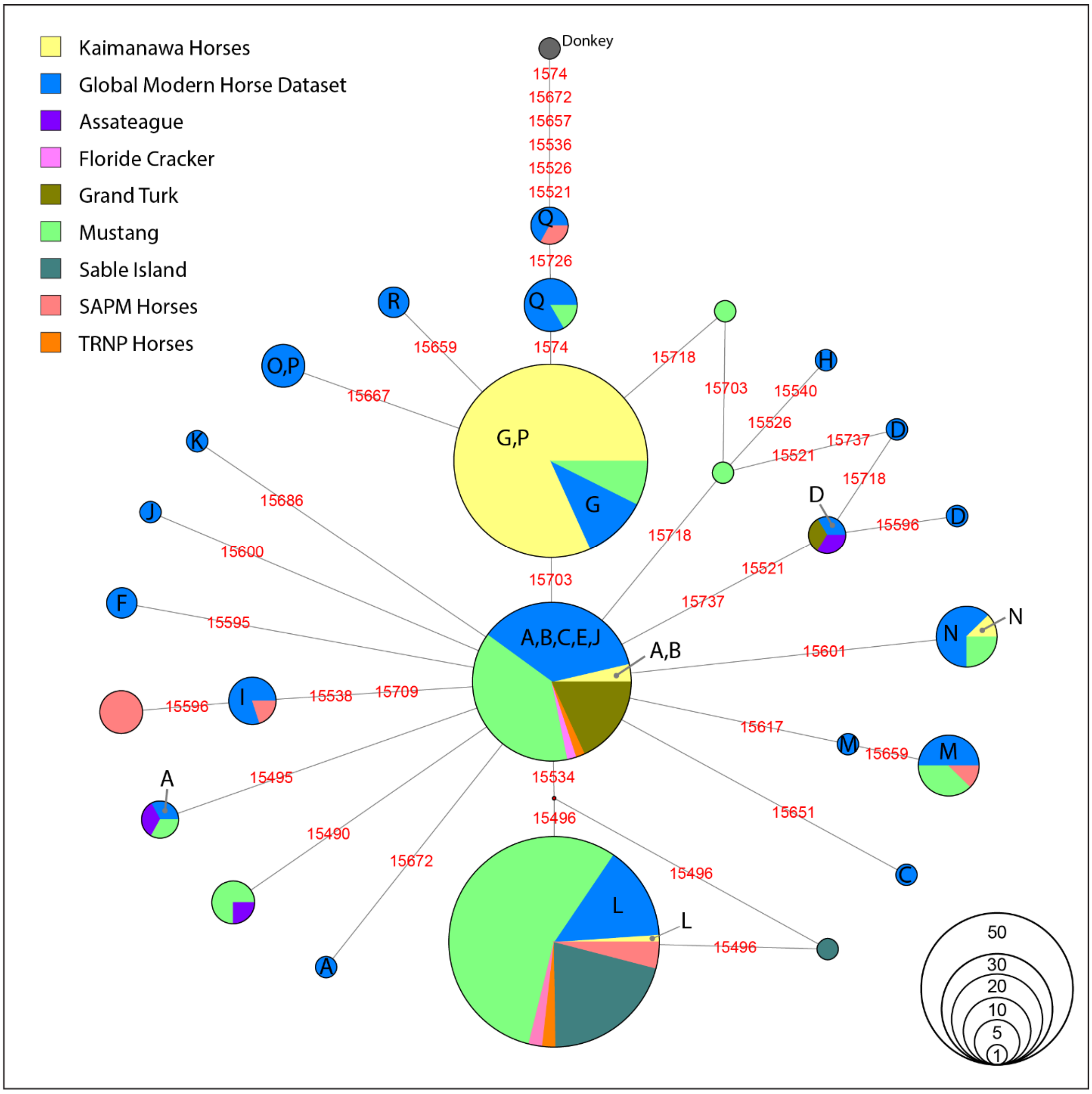

Figure S4: Partial mtDNA D-loop (225 bp) HG network in KHs (n=71) in relation to modern horses (n=226; domestic, feral and Przewalski's Horse) from previously published dataset [10,12,20,30-32] based on 28 SNPs. The nomenclature of 18 major mitochondrial HGs (A-R) are adopted from [20]. The colors indicate different breeds and datasets. The HGs are shown as circles and the circle size is proportional to their frequency. The MJ network [28] was constructed using NETWORK v10.2.0.0 software (https://www.fluxus-engineering.com, accessed on 6 October 2022).

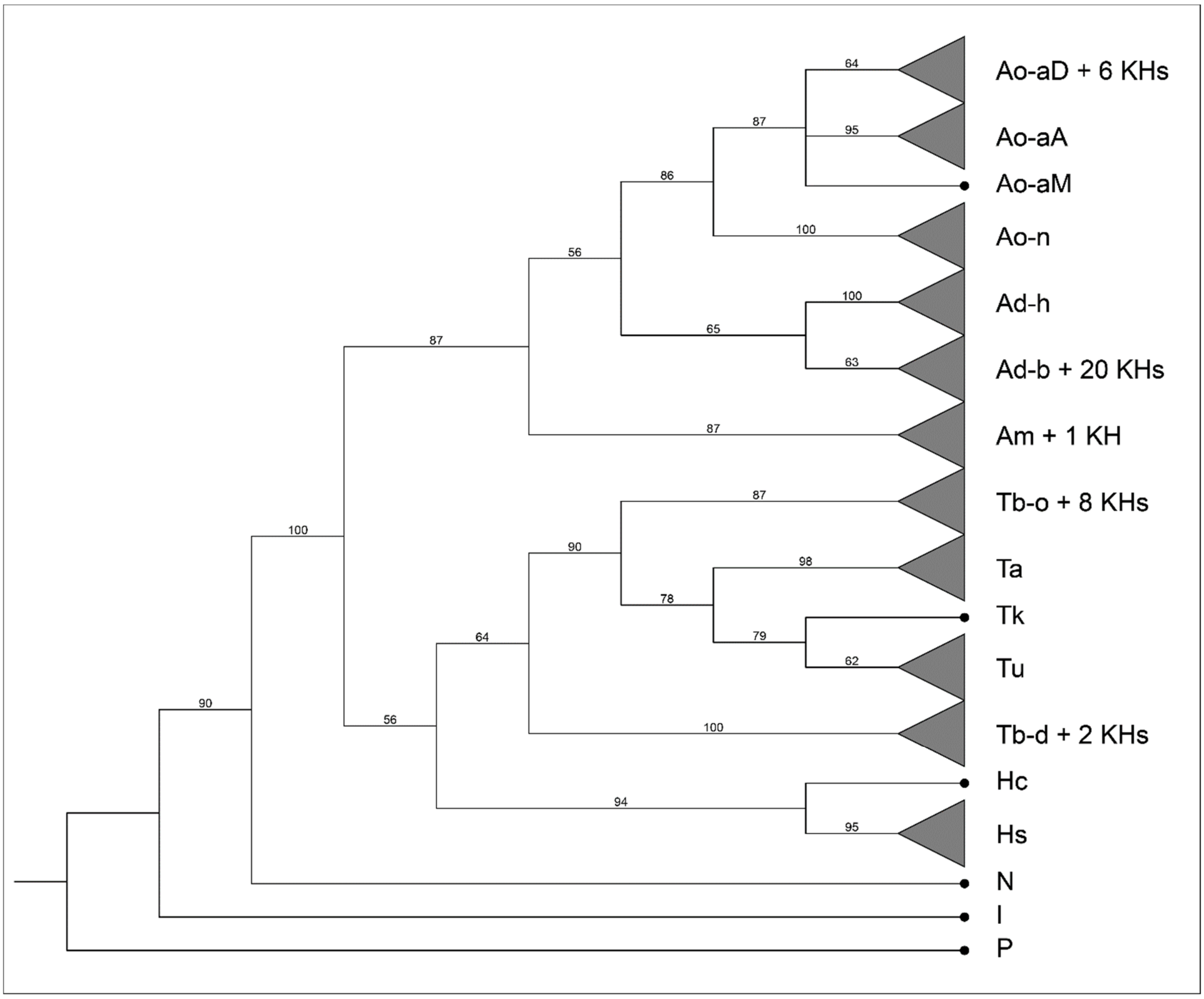

Figure S5: ML phylogenetic tree based on 157 Y-chr SNPs genotyped in KHs (n=71), and retrieved from the global modern horse breeds (n=157) [34]. The numbers on the branches indicate the bootstrap support, calculated using 1,000 replicates. The nomenclature of HGs was adopted from [33, 34] and the number of KH individuals represented by each HG are shown to the right. Przewalski horse (*Equus ferus przewalskii*) [34] was used as an outgroup and the tree was visualized using iTOL v6.4.2. [54]. ML phylogenetic tree was constructed using MEGA v.10.2.5 [27] with the K2 (Kimura-2) as the best fit substitution model based on the Bayesian Information Criterion (BIC).

5. Supplementary References

52. Katoh, K.; Standley, D.M. MAFFT Multiple Sequence Alignment Software Version 7: Improvements in Performance and Usability. *Mol. Biol. Evol.* **2013**, *30*, 772–780. https://doi.org/10.1093/molbev/mst010.
53. Hall, T.; Biosciences, I.; Carlsbad, C. BioEdit: An Important Software for Molecular Biology. *GERF Bull Biosci* **2011**, *2*, 60–61.
54. Letunic, I.; Bork, P. Interactive Tree Of Life (ITOL) v5: An Online Tool for Phylogenetic Tree Display and Annotation. *Nucleic Acids Res.* **2021**, *49*, W293–W296. https://doi.org/10.1093/nar/gkab301.
